# COVID-19 and Thromboinflammation: Is There a Role for Statins?

**DOI:** 10.6061/clinics/2021/e2518

**Published:** 2021-03-15

**Authors:** Filipe Ferrari, Vítor M. Martins, Marcelo Teixeira, Raul D. Santos, Ricardo Stein

**Affiliations:** I Programa de Pos-Graduacao em Cardiologia e Ciencias Cardiovasculares, Hospital de Clinicas de Porto Alegre, Universidade Federal do Rio Grande do Sul, Porto Alegre, RS, BR.; II Hospital de Clinicas de Porto Alegre, Porto Alegre, RS, BR.; III Hospital Israelita Albert Einstein, Sao Paulo, SP, BR.; IV Unidade Clinica de Lipides, Instituto do Coracao (InCor), Hospital das Clinicas HCFMUSP, Faculdade de Medicina, Universidade de Sao Paulo, Sao Paulo, SP, BR.; V Faculdade de Medicina, Universidade Federal do Rio Grande do Sul, Porto Alegre, RS, BR.

**Keywords:** COVID-19, Thrombosis, Inflammation, Atherosclerosis, Statins

## Abstract

The novel coronavirus disease (COVID-19) showed increased morbidity and mortality rates and worse prognosis in individuals with underlying chronic diseases, especially cardiovascular disease and its risk factors, such as hypertension, diabetes, and obesity. There is also evidence of possible links among COVID-19, myocardial infarction, and stroke. Emerging evidence suggests a pro-inflammatory milieu and hypercoagulable state in patients with this infection. Despite anticoagulation, a large proportion of patients requiring intensive care may develop life-threatening thrombotic complications. Indeed, the levels of some markers of hemostatic activation, such as D-dimer, are commonly elevated in COVID-19, indicating potential risk of deep vein thrombosis and pulmonary thromboembolism. In this review, we critically examine and discuss aspects of hypercoagulability and inflammation in COVID-19 and the possible benefits of statins in this scenario, with emphasis on their underlying molecular mechanisms. Moreover, we present recommendations on the use of antiviral drugs in combination with statins.

## INTRODUCTION

Coronaviruses have caused two epidemics in the past: severe acute respiratory syndrome (SARS) and Middle East respiratory syndrome (MERS) (1,2). However, the disease caused by severe acute respiratory syndrome coronavirus 2 (SARS-CoV-2), designated as coronavirus disease (COVID-19), has since caused an ongoing pandemic that significantly threatens global health (3). Patients infected by SARS-CoV-2 commonly have a series of comorbidities and are susceptible to a wide range of complications (1); among these, coagulation abnormalities and thromboembolic events have been frequently reported, especially in the most critically ill patients (2). In line with this, patients with acute respiratory distress syndrome (ARDS) secondary to COVID-19 are more likely to develop thrombotic complications compared to patients with ARDS of other etiologies (3). These remarkably high prevalence and incidence rates of thrombotic complications have also raised questions about an underlying hypercoagulable state in these individuals. Indeed, increased levels of D-dimer and some pro-inflammatory cytokines are frequently observed in patients with COVID-19 and have shown prognostic significance (4,5). Emerging evidence has also led to the hypothesis that these patients are at higher risk of developing disseminated intravascular coagulation (DIC) (6).

In this review, we will present and discuss clinical and laboratory findings relevant to coagulation abnormalities in patients with COVID-19, incidence of thromboembolic events, and considerations of possible interventions for the management of these complications.

### COVID-19 and Hypercoagulability

The pathogenesis of hypercoagulability in COVID-19 may be related to complement-mediated endothelial injury (7) and increased levels of circulating prothrombotic factors (8). Laboratory parameters from a small cohort of Italian patients with COVID-19 admitted to the intensive care unit (ICU) were consistent with hypercoagulability in association with a severe inflammatory state (9). In another 150 patients referred to ICUs for ARDS, 64 clinically relevant thrombotic events were diagnosed, mainly pulmonary embolism (16.7%) (3). In these patients, von Willebrand factor (vWF) activity and vWF antigen and FVIII levels were increased. More than 95% of patients had elevated D-dimer and fibrinogen levels, and 87.7% tested positive for lupus anticoagulant, a prothrombotic antibody. A reduction in platelet count has also been reported as a marker of worse prognosis, with preliminary studies showing that patients with infection usually develop thrombocytopenia (>36%) (10). In fact, a meta-analysis identified significantly lower platelet counts in patients with severe disease (weighted mean difference, -31 × 10^3^/µL; 95% confidence interval [CI], -35 to -29 ×10^3^/µL), and thrombocytopenia was associated with fivefold odds of having severe disease (odds ratio [OR], 5.1; 95% CI, 1.81 to 14.6) (11). Thrombocytopenia and elevated D-dimer levels can be explained by excessive activation of the coagulation cascade and platelets (12). D-dimer, specifically, is a product of fibrin degradation, and its elevation has been associated with a higher mortality rate, highlighting the important role played by hypercoagulability in the pathophysiology of COVID-19 (13,14). Similarly, D-dimers may stimulate the release of proinflammatory cytokines, such as interleukin 6 (IL-6) (15). These cytokines act in systemic inflammation (16), and there is evidence that elevated interleukin levels can play a role in activating coagulation and are associated with venous thromboembolism (VTE) (17). In a retrospective study, severe cases (n=11) presented higher levels of D-dimer, ferritin, and inflammatory markers, such as IL-6 and tumor necrosis factor alpha (TNF-α), than moderate cases (n=10) (18). Wang et al. (19) analyzed laboratory values of 65 patients with COVID-19 and observed that D-dimer and ferritin levels were higher in those with severe (n=20) and extremely severe (n=15) disease. In a study by Zhou et al. (20), D-dimer levels >1 μg/mL at admission were associated with an increased risk of death (OR, 18.42; 95% CI, 2.64 to 128.55). Moreover, 50% of those who died had coagulopathy compared with 7% of survivors. D-dimer levels >1000 µg/L were associated with a fatal outcome. Likewise, in another study, patients requiring ICU treatment had median D-dimer levels significantly higher than those who did not require ICU admission - 0.5 mg/L (interquartile range [IQR], 0.3-0.8) and 2.4 mg/L (IQR, 0.6-14.4), respectively (21). These findings indicate that D-dimer levels can serve as a prognostic tool for risk stratification in COVID-19.

Due to the aforementioned coagulation abnormalities, many patients with severe COVID-19 may develop life-threatening DIC (6,22). However, the DIC observed in this viral infection has distinct characteristics from that observed in sepsis. Although prolonged prothrombin time, thrombocytopenia, and increased D-dimer level are suggestive of DIC, thrombocytopenia in sepsis is usually more profound; D-dimer levels in patients with COVID-19 do not reach such high levels. Moreover, according to the International Society on Thrombosis and Haemostasis score, most patients with COVID-19 would not be classified as having DIC (4).

Some risk factors and markers of thrombosis and hemostasis in patients with COVID-19 in different studies (10,18-21,23-35) are summarized in Table 1.

### Thromboinflammation

Besides coagulation abnormalities, severe COVID-19 infection is associated with inflammatory responses, leading to acute lung injury and hypoxemic respiratory failure. This interaction between coagulation and inflammation has been termed thromboinflammation (36). Evidence suggests that patients with ARDS secondary to COVID-19 exhibit extensive systemic inflammation, presenting with an exacerbated release of pro-inflammatory cytokines and chemokines (10).

Moreover, SARS-CoV-2 infection seems to increase C-reactive protein (CRP) levels. A meta-analysis of 38 studies enrolling >3,000 patients found elevated CRP levels in 74% of patients (37). In addition, a multivariate logistic analysis indicated that high CRP levels might be an important risk factor for progression of infection (OR, 10.5; 95% CI, 1.2-34.7) (37). A study conducted in China on 27 patients with COVID-19 showed that CRP levels positively correlated with the diameter of the lung lesion and might reflect the severity of the disease (38). Similar results were observed by Tan et al. (39), who found that CRP levels predicted a more severe form of this disease. Finally, Ruan et al. (40) observed that median CRP levels in COVID-19 survivors were approximately 40 mg/L compared to 125 mg/L in nonsurvivors, suggesting a strong correlation with disease severity and prognosis.

### Thrombotic Complications in Patients with COVID-19

#### COVID-19 and Venous Thromboembolism

Abnormal coagulopathy tends to occur in most nonsurvivors of COVID-19 (31). It is known that the risk of VTE is increased in critically ill patients requiring transfer to the ICU (41). Thus, it is postulated that this risk would also be high in patients with the most severe forms of COVID-19. Complete autopsy of 12 consecutive SARS-CoV-2-positive individuals showed a high incidence of thromboembolic events (42). Specifically, postmortem examination revealed deep venous thrombosis in 7 of 12 patients (58%), while pulmonary embolism was the direct cause of death in 4 patients. A series of 107 consecutive patients with confirmed COVID-19 who were admitted for pneumonia reported a high incidence of pulmonary embolism during their ICU stay (20.6%), twice as high as the frequency found in the previous year (43). A Chinese study reported that 40% of hospitalized patients with COVID-19 had a high risk of VTE (Padua prediction score ≥4) (44). It is recommended that, in the absence of contraindications, all critically ill patients with COVID-19 receive preventive strategies for VTE (45). Despite this, in a study by Tang et al. (46), of approximately 450 patients with severe COVID-19, only slightly more than 20% received anticoagulant therapy (heparin). As some of these patients developed pulmonary embolism, this may have contributed to a higher mortality rate. In contrast, a French study examined 150 patients with COVID-19 in need of intensive treatment and compared them to patients with non-COVID ARDS. The authors observed that patients with COVID-19 developed significantly more thrombotic complications despite prophylactic or therapeutic anticoagulation (3).

A study on 143 patients hospitalized with COVID-19 showed that 66 (46%) developed deep vein thrombosis (DVT) in the lower limbs. These individuals were older and had lower oxygenation index and higher cardiac injury index. Moreover, those who developed DVT had a higher mortality rate (34.8% *versus* 11.7%, *p*=0.001). This study showed that DVT is highly prevalent and associated with adverse outcomes in hospitalized patients with COVID-19 (34).

Because of the importance of thrombotic events in patients with COVID-19, the risk of thrombosis must be carefully assessed. Monitoring platelet count, prothrombin time, activated partial thromboplastin time, and fibrinogen and D-dimer levels every 48h can be an important preventive measure. Age >70 years, prolonged bed rest, postpartum status, use of combined oral contraceptives, and obesity are factors that can contribute to increased thrombotic risk (47).

The prevalence of thromboembolic complications (e.g., DVT, VTE, and pulmonary embolism) in patients with COVID-19 across different studies (29,34,42,43,48-52) is shown in Table 2.

### Preexisting Cardiovascular Disease

Patients with preexisting cardiovascular disease have a worse prognosis if infected with SARS-CoV-2. A much higher mortality rate in patients with underlying cardiovascular disease than in the general population was reported (1), reaching almost 20% in hospitalized patients with COVID-19 (53). These patients have a higher risk of developing cardiac injury (24), being critically ill (9), and requiring intensive care (53). A retrospective series of 187 COVID-19 cases reported a 7.6% mortality rate for patients without underlying cardiovascular disease and normal troponin levels compared to almost 70% for those with cardiovascular disease and high troponin levels (24). However, it is important to note that, even with normal troponin levels, the presence of underlying cardiovascular disease is associated with a non-negligible increase in the risk of death. As an example, the study by Guo et al. (25) involving 187 patients reported a mortality rate of 13%.

Postmortem studies in Switzerland found a high prevalence of pulmonary embolism with microthrombi in the alveolar capillaries (45% of those with available histology). More than 70% of these patients had underlying chronic diseases, such as cardiovascular disease (48). Similar data were reported by Wichmann et al. (42) in autopsies of patients with COVID-19, where deep vein thrombosis and preexisting chronic diseases were both highly prevalent (58% and 50%, respectively). Although these autopsy findings should be interpreted with caution, due to the small sample size, the high incidence of thromboembolic events in individuals with preexisting cardiovascular diseases suggests a possible relationship between COVID-19-induced coagulopathy and thrombosis with these diseases.

### COVID-19 and Myocardial Infarction (MI)

The COVID-19 pandemic has caused a significant reduction in the number of cardiac interventional procedures. In Spain, for example, cardiac catheterizations fell by almost 50% (54); similar data were reported in the United States (55). The association between acute infections and increased risk of type 2 MI suggests a causal relationship. The risk of MI associated with pneumonia seems to peak at the onset of this infection and is proportional to disease severity (56). Interestingly, Kwong et al. (57) studied patients infected with influenza virus and its association with hospitalizations for MI that occurred within 1 year before or after its diagnosis. Within 7 days after detection of influenza B, influenza A, respiratory syncytial virus, and other viruses, the incidence rate of MI was >10 times (95% CI, 4.37-23.38), 5 times (95% CI, 3.02-8.84), 3.5 times (95% CI, 1.11-11.12), and 2.77 times (95% CI, 1.23-6.24) higher, respectively, than that at baseline, suggesting an association between respiratory infections and MI. A US case series has shown that the risk of MI increases exponentially during the first 15 days after hospitalization for acute bacterial pneumonia, with risk being almost 50-fold greater than in any 15-day period during the year before or after the onset of infection (58).

ST segment elevation on electrocardiogram has been reported in a series of patients with COVID-19 from New York. Importantly, although 18 patients presented with high D-dimer levels, 8 patients with clinical diagnosis of MI presented with median levels higher than those of 10 patients with non-coronary myocardial injury - 1,909 ng/mL (IQR, 682-19,653) *versus* 858 ng/mL (IQR, 541-3,580), respectively (59). In a study of patients undergoing primary percutaneous coronary intervention for ST-Elevation Myocardial Infarction (STEMI), high D-dimer levels at admission were associated with larger MI size, greater extent of area at risk, and lower myocardial salvage index, suggesting that D-dimer levels may be a marker of advanced myocardial injury (60).

What are the possible hypotheses that may explain an increased risk of MI in patients with COVID-19? With the release of pro-inflammatory cytokines and catecholamines, compounded by increases in both oxygen demand and heart rate caused by the infection, a marked reduction in coronary perfusion is observed (56). These and other factors may contribute to a mismatch between oxygen demand and oxygen supply, resulting in ischemia, injury, and fibrosis.

### COVID-19 and Obesity

Patients with COVID-19 who are overweight or obese may also be more susceptible to complications. A study enrolling 775 infected individuals showed that 72% were overweight or obese. Among those with a body mass index (BMI) >30 kg/m^2^ who were admitted to the ICU, 61% died (61). In a French retrospective study (n=124), 48% of patients were obese, and 28% were severely obese (BMI >35 kg/m^2^). As the BMI category increased, patients required more invasive mechanical ventilation (62). Similar data were observed in a study by Hu et al. (63) in China (n=58). The proportion of prolonged hospital stay was significantly higher in overweight or obese patients than in those with normal weight (62.1% *versus* 26.1%, *p*=0.01). In addition, discharge from the hospital was inversely and independently associated with BMI (hazard ratio [HR], 0.75; 95% CI, 0.63-0.90).

Some factors have been speculated to increase morbidity in obese patients with COVID-19. Increased levels of pro-inflammatory cytokines, such as TNF-α and IL-6, may be more common in overweight and obese individuals (64). This pro-inflammatory environment, in association with compromised fibrinolysis, may contribute to increased thrombotic risk, which may be associated with worsening lung damage (65). Moreover, as obese patients commonly have other comorbidities at baseline, these may represent an additional risk factor for COVID-19 complications. Finally, adipose tissue itself has been demonstrated to serve as a reservoir for several types of viruses, such as human adenovirus Ad-36, influenza A virus, and cytomegalovirus (66). By analogy, COVID-19 may also affect the adipose tissue and subsequently compromise other organs.

### Prevention of Venous Thromboembolism in Patients with COVID-19

It is known that patients with COVID-19 have an increased risk of thrombotic events (42-44). These individuals may also have increased presence of extramedullary megakaryocytes in organs such as the lungs and heart. This is a phenomenon that seems to play an important role in increasing thrombosis in these patients (67).

Klok et al. (68) studied 184 patients with COVID-19 who required ICU admission and found a 31% incidence of thrombotic complications. In turn, Tang et al. found lower 28-day mortality in heparin users - unfractionated heparin or low molecular weight heparin (LMWH) - compared to that in non-users in the subgroup with sepsis-induced coagulopathy (SIC) score ≥4 (40.0% *versus* 64.2%; *p*=0.029) or D-dimer level of more than sixfold of the upper limit of normal (32.8% *versus* 52.4%; *p*=0.017), suggesting a better prognosis in patients who met the SIC criteria or with markedly high D-dimer levels (46).

Given the clinical importance associated with a high risk of VTE in patients with severe COVID-19, antithrombotic preventive therapy is strongly recommended in the absence of contraindications (69-73). Patients with contraindications to anticoagulants should be treated with limb compression (69). Several recommendations for prophylaxis for the prevention of VTE in patients with COVID-19 were published (69-73). Many of these patients may have high Padua scores and reduced mortality rates with the use of LMWH or unfractionated heparin (72).

European authors recommend that in patients with COVID-19 and D-dimer levels <0.5 μg/mL, prophylactic-dose anticoagulation is recommended (enoxaparin 40 mg every 24h); if D-dimer level is between 0.5 and 3.0. μg/mL, enoxaparin 40 mg every 12h; if D-dimer level is >3.0 μg/mL, enoxaparin 1 mg/kg every 12h (73). Some authors suggest a more aggressive prophylactic therapy (e.g., enoxaparin 30 mg subcutaneously every 12h for those with body weight ≤120 kg and 40 mg subcutaneously every 12h for patients with body weight >120 kg) (74).

The American College of Chest Physicians recommends that hospitalized patients with COVID-19 receive anticoagulant thromboprophylaxis with LMWH or fondaparinux over unfractionated heparin; direct-acting oral anticoagulants are not indicated. In patients with severe COVID-19, standard-dose thromboprophylaxis is suggested over the intermediate one (LMWH every 12h or increased weight-based dose) (70).

The International Society on Thrombosis and Haemostasis suggests that a half-therapeutic-dose of LMWH (1 mg/kg every 24h) can be considered for high-risk patients with COVID-19 and a 50% higher dose should be considered for obese individuals (75). In addition, also recommends thromboprophylaxis with LMWH or a direct-acting oral anticoagulant (duration of 14 to 30 days) after discharge from those patients considered at high risk (e.g., age >65 years, cancer, previous VTE, elevated D-dimer level (>2 times the upper limit of normal, etc.), and low risk of bleeding (76). In contrast, thromboprophylaxis for patients who do not require hospitalization is not currently recommended (76).

In an editorial published by Lopes and Fanaroff, the authors emphasize the importance of conducting randomized clinical trials in the COVID-19 anticoagulation scenario. They pointed out that the correct dose and best type of anticoagulant for these patients must be better investigated (77).

### Possible Benefits of Statins in COVID-19

#### Anti-inflammatory and Antithrombotic Effects

**Figure 1:**
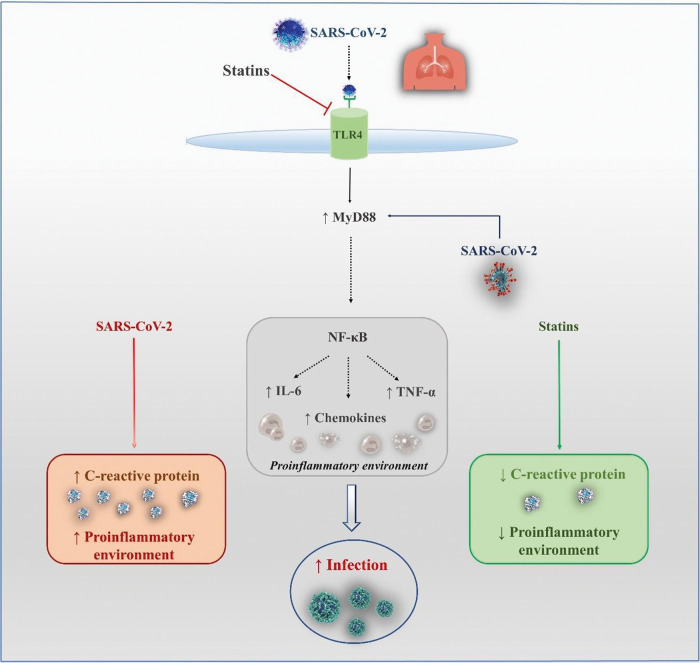
Possible protective mechanisms of statins in COVID-19. IL-6, interleukin 6; TNF-α, tumor necrosis factor alpha; NF-κB, nuclear factor kappa B.

Several mechanisms have been proposed to support the hypothesis of a protective effect of statins, as observed in studies of patients with influenza or COVID-19 (Figure 1). Reduction in inflammation via inhibition of nuclear factor kappa B (NF-κB) may be one of these components. In an experimental study (78), NF-κB inhibition led to a reduction in inflammation and lung injury and significantly increased survival after SARS-CoV-2 infection. Moreover, Bahrami et al. (79) suggested that statins could also have a positive impact on inflammatory diseases by reducing toll-like receptor 4 (TLR4) expression. Statins can also regulate the TLR4/myeloid differentiation primary response 88 (MyD88)/NF-κB signaling pathway. MyD88 is an essential adaptive molecule for signaling through TLRs, resulting in signal transduction and production of pro-inflammatory cytokines. As it has been observed that SARS-CoV infection can induce expression of the MyD88 gene, the ability of statins to maintain MyD88 at normal levels may be protective for patients with COVID-19. This balance is important because both overexpression of MyD88 and its deficiency led to increased mortality rates after MERS-CoV infection (80). However, a statin-induced increase in IL-18 levels might create an unfavorable environment in SARS-CoV-2 infection and even increase the disease severity (81).

**Figure 2:**
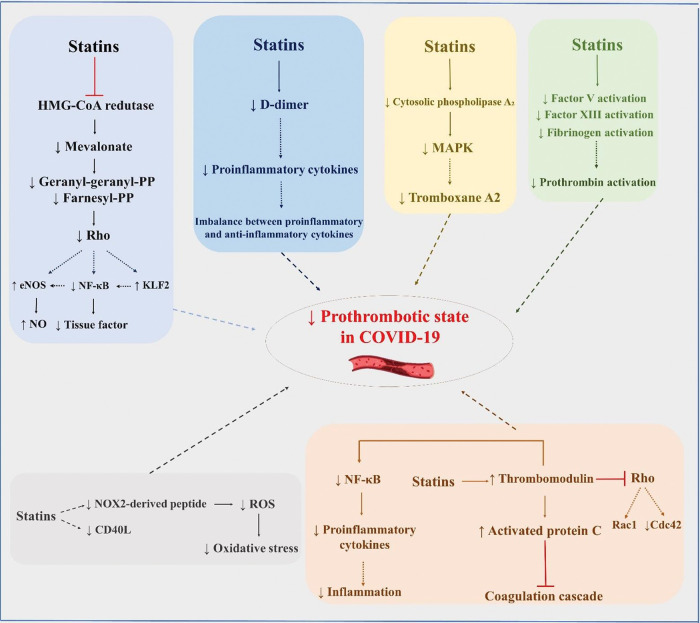
Possible pleiotropic effects of statins to reduce thrombotic complications in patients with COVID-19. eNOS, endothelial nitric oxide synthase; NF-κB, nuclear factor kappa B; KLF2, Krüppel-like factor 2; NO, nitric oxide; MAPK, mitogen-activated protein kinase; ROS, reactive oxygen species.

Statins may also exert favorable effects in COVID-19 through their anti-thrombotic effects (Figure 2). More than 20 years ago, Colli et al. (82) demonstrated that statins can have protective effects against thrombotic events. In another study, after vascular injury, early statin therapy was found to act on platelet cytosolic phospholipase A2 activity through its effects on mitogen-activated protein kinases and calcium, reducing thromboxane A2 synthesis by approximately 30% (83). An *ex vivo* study with 10 patients with hypercholesterolemia showed that, at 2h after administration of 20 mg rosuvastatin, recruitment of platelets, platelet CD40L, and soluble NOX2-derived peptide decreased, while platelet nitric oxide increased. In the group that received only dietary intervention, no change was observed. These findings suggest that rosuvastatin promotes a reduction in oxidative stress and platelet activity (84).

Bruni et al. (85) demonstrated that the administration of atorvastatin and simvastatin significantly reduced P-selectin, CD36, and LOX-1. Undas et al. (86) reported antithrombotic effects at sites of microvascular injury with a 3-month course of simvastatin in patients with coronary artery disease. Attenuation of the activity of factor V, factor XIII, fibrinogen, and prothrombin is another potential mechanism of antithrombotic action of statins, regardless of cholesterol level reduction. Statins can also increase the expression of thrombomodulin and activated protein C, inhibiting the coagulation cascade (87). Increases in mRNA levels of thrombomodulin inhibit geranylgeranylation of proteins in the Rho subfamily (e.g., Rac1/Cdc42) and, consequently, activation of NF-κB (88), promoting a less inflammatory environment. In addition to the possible pleiotropic anti-inflammatory and immunomodulatory effects of statins, which may be beneficial in COVID-19, Reiner et al. (89) suggested, based on an in *silico* molecular docking experiment, that statins may inhibit the main SARS-CoV-2 protease. This raises the hypothesis that statins may be directly beneficial against SARS-CoV-2 infection through a novel mechanism.

In 2012, Vandermeer et al. (90) included data from >3,000 patients hospitalized for influenza, one-third of whom received statin therapy. Using a multivariable logistic regression model, the authors found that statin therapy before or during hospitalization was associated with a lower risk of mortality (OR, 0.59; 95% CI, 0.38-0.92). In another study, patients with viral pneumonia (n=539) on continuous use of statins presented lower rates of death and intubation during hospitalization (OR, 0.26; 95% CI, 0.08-0.81). Therefore, patients admitted with viral respiratory diseases, including COVID-19, may benefit from therapy with these drugs. However, these findings should be interpreted with caution due to the limitations inherent to observational studies.

A meta-analysis of nine randomized clinical trials (n=1,165) showed that statins can significantly reduce plasma D-dimer levels (SMD -0.988 µg/mL; 95% CI, -1.590 to -0.385). A subgroup analysis of patients on atorvastatin and simvastatin indicated that this effect was significant only with at least 12 weeks of treatment (91). These findings suggest a potential role of statins in reducing coagulation and preventing VTE.

Zhang et al. (92) suggested a potential reduction in all-cause mortality in patients with COVID-19 who received statin therapy. In this retrospective study performed in China, approximately 14,000 patients were included, of whom 1,219 received statin therapy. Atorvastatin was the most commonly used statin (83%), followed by rosuvastatin (15%). These individuals had lower crude mortality at 28 days compared to those without statin use (incidence rate ratios, 0.78; 95% CI, 0.61-0.996). When adjustments for baseline differences were analyzed, statin therapy was also associated with lower mortality (adjusted HR, 0.63; 95% CI, 0.48-0.84; *p*=0.001). Another study observed a slower progression to death in ICU patients with COVID-19 using atorvastatin (93). Although Kow and Hasan’s meta-analysis has shown a reduction in the risk of fatal or severe disease with the use of statins (HR, 0.70; 95% CI, 0.53-0.94) compared to that with the non-use of statins in patients with COVID-19 (94), another meta-analysis did not observe improvement in disease severity (OR, 1.64; 95% CI, 0.51-5.23; *p*=0.41), nor in the mortality rate by COVID-19 (OR, 0.78; 95% CI, 0.50-1.21; *p*=0.26) (95).

Despite indirect evidence pointing to the potential benefit of statins in patients with COVID-19, there is a lack of robust clinical evidence on their role in reducing clinical outcomes.

### Prevention of Venous Thromboembolism with Statins

VTE has a significant impact on the patient’s health costs, morbidity, and mortality. In the absence of adequate medical treatment, VTE can reduce blood flow and oxygen demand, damaging organs and tissues (96).

In a prespecified analysis of the JUPITER trial, the authors evaluated the impact of rosuvastatin on the first occurrence of VTE. During a median follow-up of 1.9 years (maximum 5.0), there were 34 occurrences of symptomatic VTE in the rosuvastatin group and 60 in the placebo group, corresponding to a 43% reduction in the VTE rates in favor of the statin group (HR, 0.57; 95% CI, 0.37-0.86; *p*=0.007) (97). Stewart et al. (98) evaluated the impact of statin treatment on VTE recurrence in a cohort of 192,908 patients (mean age, 67 years) recruited between 2004 and 2017. After propensity matching, the group of patients who did not use statins presented higher rates of VTE recurrence compared to statin users (20% *versus* 16%; *p*<0.0001). After adjustments for a series of variables, a 25% reduction in VTE recurrence was observed in patients receiving statin (OR, 0.75; 95% CI, 0.72-0.79).

These data were analyzed by meta-analyses. In one of them, in the risk analysis of VTE development (one randomized clinical trial and eight observational studies; n=845.445), it was reported that the use of statins reduced the odds of developing VTE by 32% (adjusted OR, 0.68; 95% CI, 0.54-0.86) (99). In a subsequent meta-analysis, Kunutsor et al. (100) compiled 13 cohort studies (n=3,148,259) and 23 randomized clinical trials (n=118,464) and analyzed them separately. The authors found a reduction in the risk of VTE when the statin group was compared to the control group (relative risk [RR], 0.75; 95% CI, 0.65-0.87; *p*<0.0001; 0.85; 95% CI, 0.73-0.99; *p*=0.038, respectively). In addition, rosuvastatin showed greater benefit over the risk of VTE compared to other statins (RR, 0.57; 95% CI, 0.22-0.75; *p*=0.015).

These data should be viewed with caution given their potential biases (101), and randomized clinical trials are needed to assess the real effectiveness of statins in VTE prevention.

### Drug-Drug Interactions with Antiviral Therapy

It is important to mention that caution should be taken when administering statins (102) concomitantly with antiviral therapy in patients with COVID-19 (Table 3).

## CONCLUSIONS

COVID-19 is associated with a hyperinflammatory and hypercoagulable state, especially in patients with severe disease, which results in an increased risk of thrombotic complications. Patients with preexisting cardiovascular disease and those with cardiovascular risk factors seem to be at high risk for severe complications of SARS-CoV-2 infection. Although statins have established anti-inflammatory and antithrombotic effects, there is no clear evidence that these drugs reduce morbidity and mortality in patients with COVID-19. Therefore, their use needs to be further explored through the conduction of high-quality randomized clinical trials.

**Conflicts of Interest.** The author RDS has received honoraria for consulting, research, and/or speaker activities from Aché, Amgen, AstraZeneca, Esperion, Kowa, Novo Nordisk, Merck, MSD, Pfizer, PTC, and Sanofi/Regeneron.

## AUTHOR CONTRIBUTIONS

Ferrari F, Martins VM, Santos RD and Stein R were responsible for the research design and conception. Ferrari F, Martins VM, Teixeira M, Santos RD and Stein R were responsible for the data acquisition, analysis and interpretation, and manuscript writing. Ferrari F, Santos RD and Stein R were responsible for the critical revision of the manuscript on important intellectual content.

## Figures and Tables

**Table 1:** Risk factors and characteristics of coagulation and hemostasis markers in patients with COVID-19.

Studies	N	HTN / CVD (%)	D-dimer (mg/L) (IQR)	Prothrombin time (s) (IQR)	Platelet count (×10^3^/µL) (IQR)
Chen et al. (18)	21	Severe: 36.4 / NA Moderate: 10 / NA	Severe: 2.6 (0.6 to 18.7) Moderate: 0.3 (0.3 to 0.4)	Severe: 14.3 (13.6 to 14.6) Moderate: 13.4 (12.8 to 13.7)	Severe: 157 (134 to 184.5) Moderate: 175.6 (148.3 to 194)
Fraissé et al. (24)	92	TEV: 62 / 3 No TEV: 65 / 14	With TEV: 4.4 (1.8 to 2) Without TEV: 2.2 (1.2 to 5.9)	NA	NA
Guan et al. (10)	1.099	Severe: 23.7 / 5.8 Nonsevere: 13.4 / 1.8	NA	NA	Severe: 137.5 (99 to 179)^ϕ^ Nonsevere: 172 (139 to 212)^ϕ^
Guo et al. (25)	187	Normal TnT: 20.7 / 3 Elevated TnT: 63.5 / 48.3	Normal TnT: 0.29 (0.17 to 0.6) Elevated TnT: 3.85 (0.51 to 25.28)	Normal TnT: 12.4 (12 to 13) Elevated TnT: 13.3 (12.2 to 15.3)	NA
Han et al. (26)	94	NA	Severe: 19.1 Nonsevere: 2.1	Severe: 12.7 Nonsevere: 12.2	NA
Huang et al. (21)	41	ICU: 15 / 23 Non-ICU: 14 / 11	ICU: 2.4 (0.6 to 14.4) Non-ICU: 0.5 (0.3 to 0.8)	ICU: 12.2 (11.2 to 13.4) Non-ICU: 10.7 (9.8 to 12.1)	ICU: 196.0 (165 to 263) Non-ICU: 149.0 (131 to 263)
Khamis et al. (27)	63	ICU: 29 / 8.3 Non-ICU: 33 / 5.1	ICU: 2.3 (0.7 to 4.3) Non-ICU: 0.4 (0.2 to 0.7)	NA	ICU: 253 (213 to 322) Non-ICU: 215 (169 to 264)
Liu et al. (28)	78	Progression: 9 / NA Improvement/stabilization: 18.2 / NA	Progression: 0.56 (0.21 to 6.84)* Improvement/stabilization: 0.39 (0.2 to 1.07)*	NA	Progression: 143.9 (64.81^†^) Improvement/stabilization: 173.2 (55.37^†^)
Pan et al. (29)	124	Discharge: 42.9 / 17.1 Nonsurvivor: 52.8 / 14.6	Discharge: 1.12 (0.43 to 5.42) Nonsurvivor: 3.97 (0.78 to 8)	NA	Discharge: 197 (143 to 234) Nonsurvivor: 182 (151 to 229)
Ren et al. (30)	48	All: 39.6 / 22.9	All: 3.48 (0.83 to 9.23)	NA	NA
Suleyman et al. (31)	355	ICU: 78.7 / 35 Non-ICU: 68.7 / 24.8	ICU: 1.43 (0.81 to 3.22) Non-ICU: 0.95 (0.57 to 1.57)	NA	NA
Tang et al. (32)	183	NA	Nonsurvivor: 2.1 (0.8 to 5.3) Survivor: 0.6 (0.4 to 1.3)	Nonsurvivor: 15.5 (14.4 to 16.3) Survivor: 13.6 (13 to 14)	NA
Wang et al. (19)	65	NA	Mild: 1.6 (3^†^) Severe: 4.7 (7.4^†^) Extremely severe: 6.9 (8.4^†^)	NA	NA
Wang et al. (23)	138	ICU: 58.3 / 25.0 No ICU: 21.6 / 10.8	ICU: 414 (191 to 1324) Non-ICU: 166 (101 to 285)	ICU: 13.2 (12.3 to 14.5) Non-ICU: 12.9 (12.3 to 13.4)	ICU: 142 (119 to 202) Non-ICU: 165 (125 to 188)
Wu et al. (33)	201	Nonsurvivor: 36.4 / 9.1 Survivor: 17.5 / 2.5	Nonsurvivor: 3.95 (1.15 to 10.96) Survivor: 0.49 (0.31 to 1.18)	Nonsurvivor: 11.6 (11.1 to 12.45) Survivor: 11.75 (10.95 to 12.45)	Nonsurvivor: 162 (110.5 to 231) Survivor: 204 (137.25 to 262.75)
Yang et al. (34)	52	Nonsurvivor: NA / 9 Survivor: NA / 10	NA	Nonsurvivor: 12.9 (2.9) Survivor: 10.9 (2.7)	Nonsurvivor: 191 (74^†^) Survivor: 164 (63^†^)
Zhang et al. (35)	143	DVT: 42.2 / 13.6 Non-DVT: 36.4 / 10.4	DVT: 6.6 (2.5 to 8) Non-DVT: 0.9 (0.4 to 3.5)	DVT: 14.2 (13.3 to 15.4) Non-DVT: 12.9 (12.3 to 14)	DVT: 185.5 (140.3 to 289) Non-DVT: 229 (170.5 to 285.8)
Zhou et al. (20)	191	Nonsurvivor: 48 / 24 Survivor: 23 / 1	Nonsurvivor: 5.2 (1.5 to 21.1) Survivor: 0.6 (0.3 to 1.0)	Nonsurvivor: 12.1 (11.2 to 13.7) Survivor: 11.4 (10.4 to 12.6)	Nonsurvivor: 0.6 (0.5 to 0.8) Survivor: 1.1 (0.8 to 1.5)

HTN, hypertension; CVD, cardiovascular disease; DVT, deep vein thrombosis; TEV, thrombotic events; TnT, troponin T; ICU, intensive care unit; IQR, interquartile range; NA, not available. ^†^Standard deviation.^ ϕ^Median. *ng/mL.

**Table 2:** Prevalence of thromboembolic complications in patients with COVID-19.

Studies	N	Deep Vein Thrombosis - N (%)	Venous Thromboembolism - N (%)	Pulmonary Embolism - N (%)
Cui et al. (48)	81	NA	Critically ill: 20 (16%)	NA
Menter et al. (49)	21	NA	NA	Died: 4 (19%)
Middeldorp et al. (50)	198	NA	All: 19 (20%) Critically ill: 35 (47%) Non-critically ill: 4 (3%)	All: 13 (7%) Critically ill: 11 (15%) Non-critically ill: 4 (2%)
Nahum et al. (51)	34	Critically ill at admission: 22 (65%) Critically ill 48h after admission: 5 (15%)	NA	NA
Poissy et al. (43)	107	NA	NA	Critically ill: 22 (21%)
Ren et al. (30)	48	Critically ill: 41 (85%)	NA	NA
Voicu et al. (52)	56	Invasive mechanical ventilation: 20 (36%)	NA	NA
Wichmann et al. (42)	12	Died: 7 (58%)	NA	NA
Zhang et al. (35)	143	All: 66 (46%)	NA	NA

NA: Not available.

**Table 3:** Concomitant use of antivirals and statins in patients with COVID-19.

Drug	Use with lopinavir/ritonavir	Rationale
*Atorvastatin*	Can be administered	Prefer the lowest possible dose
*Lovastatin*	Contraindicated	Increased risk of rhabdomyolysis
*Rosuvastatin*	Can be administered	Prefer the lowest possible dose
*Simvastatin*	Contraindicated	Increased risk of rhabdomyolysis

CYP450, cytochrome P450; BID, twice daily.

## References

[1] Wu Z, McGoogan JM (2020). Characteristics of and Important Lessons From the Coronavirus Disease 2019 (COVID-19) Outbreak in China: Summary of a Report of 72314 Cases From the Chinese Center for Disease Control and Prevention. JAMA.

[2] Al-Ani F, Chehade S, Lazo-Langner A (2020). Thrombosis risk associated with COVID-19 infection. A scoping review. Thromb Res.

[3] Helms J, Tacquard C, Severac F, Leonard-Lorant I, Ohana M, Delabranche X (2020). High risk of thrombosis in patients with severe SARS-CoV-2 infection: a multicenter prospective cohort study. Intensive Care Med.

[4] Levi M, Thachil J, Iba T, Levy JH (2020). Coagulation abnormalities and thrombosis in patients with COVID-19. Lancet Haematol.

[5] The Lancet Haematology (2020). COVID-19 coagulopathy: an evolving story. Lancet Haematol.

[6] Becker RC (2020). COVID-19 update: Covid-19-associated coagulopathy. J Thromb Thrombolysis.

[7] Magro C, Mulvey JJ, Berlin D, Nuovo G, Salvatore S, Harp J (2020). Complement associated microvascular injury and thrombosis in the pathogenesis of severe COVID-19 infection: A report of five cases. Transl Res.

[8] Ranucci M, Ballotta A, Di Dedda U, Bayshnikova E, Dei Poli M, Resta M (2020). The procoagulant pattern of patients with COVID-19 acute respiratory distress syndrome. J Thromb Haemost.

[9] Panigada M, Bottino N, Tagliabue P, Grasselli G, Novembrino C, Chantarangkul V (2020). Hypercoagulability of COVID-19 patients in intensive care unit. A report of thromboelastography findings and other parameters of hemostasis. J Thromb Haemost.

[10] Guan WJ, Ni ZY, Hu Y, Liang WH, Ou CQ, He JX (2020). Clinical Characteristics of Coronavirus Disease 2019 in China. N Engl J Med.

[11] Lippi G, Plebani M, Henry BM (2020). Thrombocytopenia is associated with severe coronavirus disease 2019 (COVID-19) infections: A meta-analysis. Clin Chim Acta.

[12] Giannis D, Ziogas IA, Gianni P (2020). Coagulation disorders in coronavirus infected patients: COVID-19, SARS-CoV-1, MERS-CoV and lessons from the past. J Clin Virol.

[13] Zhang L, Yan X, Fan Q, Liu H, Liu X, Liu Z (2020). D-dimer levels on admission to predict in-hospital mortality in patients with Covid-19. J Thromb Haemost.

[14] Garcia-Olivé I, Sintes H, Radua J, Abad Capa J, Rosell A (2020). D-dimer in patients infected with COVID-19 and suspected pulmonary embolism. Respir Med.

[15] Lowe GD, Rumley A, McMahon AD, Ford I, O'Reilly DS, Packard CJ (2004). Interleukin-6, fibrin D-dimer, and coagulation factors VII and XIIa in prediction of coronary heart disease. Arterioscler Thromb Vasc Biol.

[16] Bester J, Pretorius E (2016). Effects of IL-1β, IL-6 and IL-8 on erythrocytes, platelets and clot viscoelasticity. Sci Rep.

[17] Vormittag R, Hsieh K, Kaider A, Minar E, Bialonczyk C, Hirschl M (2006). Interleukin-6 and interleukin-6 promoter polymorphism (-174) G > C in patients with spontaneous venous thromboembolism. Thromb Haemost.

[18] Chen G, Wu D, Guo W, Cao Y, Huang D, Wang H (2020). Clinical and immunological features of severe and moderate coronavirus disease 2019. J Clin Invest.

[19] Wang F, Hou H, Luo Y, Tang G, Wu S, Huang M (2020). The laboratory tests and host immunity of COVID-19 patients with different severity of illness. JCI Insight.

[20] Zhou F, Yu T, Du R, Fan G, Liu Y, Liu Z (2020). Clinical course and risk factors for mortality of adult inpatients with COVID-19 in Wuhan, China: a retrospective cohort study. Lancet.

[21] Huang C, Wang Y, Li X, Ren L, Zhao J, Hu Y (2020). Clinical features of patients infected with 2019 novel coronavirus in Wuhan, China. Lancet.

[22] Terpos E, Ntanasis-Stathopoulos I, Elalamy I, Kastritis E, Sergentanis TN, Politou M (2020). Hematological findings and complications of COVID-19. Am J Hematol.

[23] Wang D, Hu B, Hu C, Zhu F, Liu X, Zhang J (2020). Clinical Characteristics of 138 Hospitalized Patients With 2019 Novel Coronavirus-Infected Pneumonia in Wuhan, China. JAMA.

[24] Fraissé M, Logre E, Pajot O, Mentec H, Plantefàve G, Contou D (2020). Thrombotic and hemorrhagic events in critically ill COVID-19 patients: a French monocenter retrospective study. Crit Care.

[25] Guo T, Fan Y, Chen M, Wu X, Zhang L, He T (2020). Cardiovascular Implications of Fatal Outcomes of Patients With Coronavirus Disease 2019 (COVID-19). JAMA Cardiol.

[26] Han H, Yang L, Liu R, Liu F, Wu KL, Li J (2020). Prominent changes in blood coagulation of patients with SARS-CoV-2 infection. Clin Chem Lab Med.

[27] Khamis F, Al-Zakwani I, Al Naamani H, Al Lawati S, Pandak N, Omar MB (2020). Clinical characteristics and outcomes of the first 63 adult patients hospitalized with COVID-19: An experience from Oman. J Infect Public Health.

[28] Liu W, Tao ZW, Wang L, Yuan ML, Liu K, Zhou L (2020). Analysis of factors associated with disease outcomes in hospitalized patients with 2019 novel coronavirus disease. Chin Med J (Engl).

[29] Pan F, Yang L, Li Y, Liang B, Li L, Ye T (2020). Factors associated with death outcome in patients with severe coronavirus disease-19 (COVID-19): a case-control study. Int J Med Sci.

[30] Ren B, Yan F, Deng Z, Zhang S, Xiao L, Wu M (2020). Extremely High Incidence of Lower Extremity Deep Venous Thrombosis in 48 Patients With Severe COVID-19 in Wuhan. Circulation.

[31] Suleyman G, Fadel RA, Malette KM, Hammond C, Abdulla H, Entz A (2020). Clinical Characteristics and Morbidity Associated With Coronavirus Disease 2019 in a Series of Patients in Metropolitan Detroit. JAMA Netw Open.

[32] Tang N, Li D, Wang X, Sun Z (2020). Abnormal coagulation parameters are associated with poor prognosis in patients with novel coronavirus pneumonia. J Thromb Haemost.

[33] Wu C, Chen X, Cai Y, Xia J, Zhou X, Xu S (2020). Risk Factors Associated With Acute Respiratory Distress Syndrome and Death in Patients With Coronavirus Disease 2019 Pneumonia in Wuhan, China. JAMA Intern Med.

[34] Yang X, Yu Y, Xu J, Shu H, Xia J, Liu H (2020). Clinical course and outcomes of critically ill patients with SARS-CoV-2 pneumonia in Wuhan, China: a single-centered, retrospective, observational study. Lancet Respir Med.

[35] Zhang L, Feng X, Zhang D, Jiang C, Mei H, Wang J (2020). Deep Vein Thrombosis in Hospitalized Patients With COVID-19 in Wuhan, China: Prevalence, Risk Factors, and Outcome. Circulation.

[36] Connors JM, Levy JH (2020). Thromboinflammation and the hypercoagulability of COVID-19. J Thromb Haemost.

[37] Zhu J, Ji P, Pang J, Zhong Z, Li H, He C (2020). Clinical characteristics of 3062 COVID-19 patients: A meta-analysis. J Med Virol.

[38] Wang L (2020). C-reactive protein levels in the early stage of COVID-19. Med Mal Infect.

[39] Tan C, Huang Y, Shi F, Tan K, Ma Q, Chen Y (2020). C-reactive protein correlates with computed tomographic findings and predicts severe COVID-19 early. J Med Virol.

[40] Ruan Q, Yang K, Wang W, Jiang L, Song J (2020). Clinical predictors of mortality due to COVID-19 based on an analysis of data of 150 patients from Wuhan, China. Intensive Care Med.

[41] Minet C, Potton L, Bonadona A, Hamidfar-Roy R, Somohano CA, Lugosi M (2015). Venous thromboembolism in the ICU: main characteristics, diagnosis and thromboprophylaxis. Crit Care.

[42] Wichmann D, Sperhake JP, Lütgehetmann M, Steurer S, Edler C, Heinemann A (2020). Autopsy Findings and Venous Thromboembolism in Patients With COVID-19: A Prospective Cohort Study. Ann Intern Med.

[B43] Poissy J, Goutay J, Caplan M, Parmentier E, Duburcq T, Lassalle F (2020). Pulmonary Embolism in Patients With COVID-19: Awareness of an Increased Prevalence. Circulation.

[44] Wang T, Chen R, Liu C, Liang W, Guan W, Tang R (2020). Attention should be paid to venous thromboembolism prophylaxis in the management of COVID-19. Lancet Haematol.

[45] Zhai Z, Li C, Chen Y, Gerotziafas G, Zhang Z, Wan J (2020). Prevention and Treatment of Venous Thromboembolism Associated with Coronavirus Disease 2019 Infection: A Consensus Statement before Guidelines. Thromb Haemost.

[46] Tang N, Bai H, Chen X, Gong J, Li D, Sun Z (2020). Anticoagulant treatment is associated with decreased mortality in severe coronavirus disease 2019 patients with coagulopathy. J Thromb Haemost.

[47] Susen S, Tacquard CA, Godon A, Mansour A, Garrigue D, Nguyen P (2020). Prevention of thrombotic risk in hospitalized patients with COVID-19 and hemostasis monitoring. Crit Care.

[48] Cui S, Chen S, Li X, Liu S, Wang F (2020). Prevalence of venous thromboembolism in patients with severe novel coronavirus pneumonia. J Thromb Haemost.

[49] Menter T, Haslbauer JD, Nienhold R, Savic S, Hopfer H, Deigendesch N (2020). Postmortem examination of COVID19 patients reveals diffuse alveolar damage with severe capillary congestion and variegated findings in lungs and other organs suggesting vascular dysfunction. Histopathology.

[50] Middeldorp S, Coppens M, van Haaps TF, Foppen M, Vlaar AP, Müller MCA (2020). Incidence of venous thromboembolism in hospitalized patients with COVID-19. J Thromb Haemost.

[51] Nahum J, Morichau-Beauchant T, Daviaud F, Echegut P, Fichet J, Maillet JM (2020). Venous Thrombosis Among Critically Ill Patients With Coronavirus Disease 2019 (COVID-19). JAMA Netw Open.

[52] Voicu S, Bonnin P, Stépanian A, Chousterman BG, Le Gall A, Malissin I (2020). High Prevalence of Deep Vein Thrombosis in Mechanically Ventilated COVID-19 Patients. J Am Coll Cardiol.

[53] Shi S, Qin M, Shen B, Cai Y, Liu T, Yang F (2020). Association of Cardiac Injury With Mortality in Hospitalized Patients With COVID-19 in Wuhan, China. JAMA Cardiol.

[54] Rodríguez-Leor O, Alvarez-Álvarez B, Ojeda S, Martín-Moreiras J, Rumoroso J, López-Palop R (2020). Impacto de la pandemia de COVID-19 sobre la actividad asistencial en cardiología intervencionista en Espaãa. REC Interv Cardiol.

[55] Garcia S, Albaghdadi MS, Meraj PM, Schmidt C, Garberich R, Jaffer FA (2020). Reduction in ST-Segment Elevation Cardiac Catheterization Laboratory Activations in the United States During COVID-19 Pandemic. J Am Coll Cardiol.

[56] Musher DM, Abers MS, Corrales-Medina VF (2019). Acute Infection and Myocardial Infarction. N Engl J Med.

[57] Kwong JC, Schwartz KL, Campitelli MA, Chung H, Crowcroft NS, Karnauchow T (2018). Acute Myocardial Infarction after Laboratory-Confirmed Influenza Infection. N Engl J Med.

[58] Corrales-Medina VF, Serpa J, Rueda AM, Giordano TP, Bozkurt B, Madjid M (2009). Acute bacterial pneumonia is associated with the occurrence of acute coronary syndromes. Medicine (Baltimore).

[59] Bangalore S, Sharma A, Slotwiner A, Yatskar L, Harari R, Shah B (2020). ST-Segment Elevation in Patients with Covid-19 - A Case Series. N Engl J Med.

[60] Choi S, Jang WJ, Song YB, Lima JA, Guallar E, Choe YH (2016). D-Dimer Levels Predict Myocardial Injury in ST-Segment Elevation Myocardial Infarction: A Cardiac Magnetic Resonance Imaging Study. PLoS One.

[61] Muscogiuri G, Pugliese G, Barrea L, Savastano S, Colao A (2020). Commentary: Obesity: The “Achilles heel” for COVID-19?. Metabolism.

[62] Simonnet A, Chetboun M, Poissy J, Raverdy V, Noulette J, Duhamel A (2020). High Prevalence of Obesity in Severe Acute Respiratory Syndrome Coronavirus-2 (SARS-CoV-2) Requiring Invasive Mechanical Ventilation. Obesity (Silver Spring).

[63] Hu X, Pan X, Zhou W, Gu X, Shen F, Yang B (2020). Clinical epidemiological analyses of overweight/obesity and abnormal liver function contributing to prolonged hospitalization in patients infected with COVID-19. Int J Obes (Lond).

[64] Schmidt FM, Weschenfelder J, Sander C, Minkwitz J, Thormann J, Chittka T (2015). Inflammatory cytokines in general and central obesity and modulating effects of physical activity. PLoS One.

[65] Goodman RB, Pugin J, Lee JS, Matthay MA (2003). Cytokine-mediated inflammation in acute lung injury. Cytokine Growth Factor Rev.

[66] Bourgeois C, Gorwood J, Barrail-Tran A, Lagathu C, Capeau J, Desjardins D (2019). Specific Biological Features of Adipose Tissue, and Their Impact on HIV Persistence. Front Microbiol.

[67] Ortega-Paz L, Capodanno D, Montalescot G, Angiolillo DJ (2021). Coronavirus Disease 2019-Associated Thrombosis and Coagulopathy: Review of the Pathophysiological Characteristics and Implications for Antithrombotic Management. J Am Heart Assoc.

[68] Klok FA, Kruip MJHA, van der Meer NJM, Arbous MS, Gommers DAMPJ, Kant KM (2020). Incidence of thrombotic complications in critically ill ICU patients with COVID-19. Thromb Res.

[69] Marietta M, Ageno W, Artoni A, De Candia E, Gresele P, Marchetti M (2020). COVID-19 and haemostasis: a position paper from Italian Society on Thrombosis and Haemostasis (SISET). Blood Transfus.

[70] Moores LK, Tritschler T, Brosnahan S, Carrier M, Collen JF, Doerschug K (2020). Prevention, Diagnosis, and Treatment of VTE in Patients With Coronavirus Disease 2019: CHEST Guideline and Expert Panel Report. Chest.

[B71] Sociedad Espaãola de Trombosis y Hemostasia. 2020 Recomendaciones de tromboprofilaxis y tratamiento antitrombótico en pacientes con COVID-19.

[B72] Aryal MR, Gosain R, Donato A, Pathak R, Bhatt VR, Katel A (2020). Venous Thromboembolism in COVID-19: Towards an Ideal Approach to Thromboprophylaxis, Screening, and Treatment. Curr Cardiol Rep.

[73] Atallah B, Mallah SI, AlMahmeed W (2020). Anticoagulation in COVID-19. Eur Heart J Cardiovasc Pharmacother.

[74] McBane RD 2nd, Torres Roldan VD, Niven AS, Pruthi RK, Franco PM, Linderbaum JA (2020). Anticoagulation in COVID-19: A Systematic Review, Meta-analysis, and Rapid Guidance From Mayo Clinic. Mayo Clin Proc.

[75] Spyropoulos AC, Levy JH, Ageno W, Connors JM, Hunt BJ, Iba T (2020). Scientific and Standardization Committee communication: Clinical guidance on the diagnosis, prevention, and treatment of venous thromboembolism in hospitalized patients with COVID-19. J Thromb Haemost.

[76] Piazza G, Morrow DA (2020). Diagnosis, Management, and Pathophysiology of Arterial and Venous Thrombosis in COVID-19. JAMA.

[77] Lopes RD, Fanaroff AC (2020). Anticoagulation in COVID-19. It Is Time for High-Quality Evidence. J Am Coll Cardiol.

[78] DeDiego ML, Nieto-Torres JL, Regla-Nava JA, Jimenez-Guardeão JM, Fernandez-Delgado R, Fett C (2014). Inhibition of NF-κB-mediated inflammation in severe acute respiratory syndrome coronavirus-infected mice increases survival. J Virol.

[79] Bahrami A, Parsamanesh N, Atkin SL, Banach M, Sahebkar A (2018). Effect of statins on toll-like receptors: a new insight to pleiotropic effects. Pharmacol Res.

[80] Yuan S (2015). Statins May Decrease the Fatality Rate of Middle East Respiratory Syndrome Infection. mBio.

[81] Rogers AJ, Guan J, Trtchounian A, Hunninghake GM, Kaimal R, Desai M (2019). Association of Elevated Plasma Interleukin-18 Level With Increased Mortality in a Clinical Trial of Statin Treatment for Acute Respiratory Distress Syndrome. Crit Care Med.

[82] Colli S, Eligini S, Lalli M, Camera M, Paoletti R, Tremoli E (1997). Vastatins inhibit tissue factor in cultured human macrophages. A novel mechanism of protection against atherothrombosis. Arterioscler Thromb Vasc Biol.

[83] Moscardó A, Vallés J, Latorre A, Madrid I, Santos MT (2013). Reduction of platelet cytosolic phospholipase A2 activity by atorvastatin and simvastatin: biochemical regulatory mechanisms. Thromb Res.

[84] Pignatelli P, Carnevale R, Pastori D, Cangemi R, Napoleone L, Bartimoccia S (2012). Immediate antioxidant and antiplatelet effect of atorvastatin via inhibition of Nox2. Circulation.

[85] Bruni F, Pasqui AL, Pastorelli M, Bova G, Cercignani M, Palazzuoli A (2005). Different effect of statins on platelet oxidized-LDL receptor (CD36 and LOX-1) expression in hypercholesterolemic subjects. Clin Appl Thromb Hemost.

[86] Undas A, Brummel KE, Musial J, Mann KG, Szczeklik A (2001). Simvastatin depresses blood clotting by inhibiting activation of prothrombin, factor V, and factor XIII and by enhancing factor Va inactivation. Circulation.

[87] Undas A, Brummel-Ziedins KE, Mann KG (2005). Statins and blood coagulation. Arterioscler Thromb Vasc Biol.

[88] Violi F, Calvieri C, Ferro D, Pignatelli P (2013). Statins as antithrombotic drugs. Circulation.

[89] Reiner Ž, Hatamipour M, Banach M, Pirro M, Al-Rasadi K, Jamialahmadi T (2020). Statins and the COVID-19 main protease: *in silico* evidence on direct interaction. Arch Med Sci.

[90] Vandermeer ML, Thomas AR, Kamimoto L, Reingold A, Gershman K, Meek J (2012). Association between use of statins and mortality among patients hospitalized with laboratory-confirmed influenza virus infections: a multistate study. J Infect Dis.

[91] Sahebkar A, Serban C, Mikhailidis DP, Undas A, Lip GY, Muntner P (2015). Association between statin use and plasma D-dimer levels. A systematic review and meta-analysis of randomised controlled trials. Thromb Haemost.

[92] Zhang XJ, Qin JJ, Cheng X, Shen L, Zhao YC, Yuan Y (2020). In-Hospital Use of Statins Is Associated with a Reduced Risk of Mortality among Individuals with COVID-19. Cell Metab.

[93] Rodriguez-Nava G, Trelles-Garcia DP, Yanez-Bello MA, Chung CW, Trelles-Garcia VP, Friedman HJ (2020). Atorvastatin associated with decreased hazard for death in COVID-19 patients admitted to an ICU: a retrospective cohort study. Crit Care.

[94] Kow CS, Hasan SS (2020). Meta-analysis of Effect of Statins in Patients with COVID-19. Am J Cardiol.

[95] Hariyanto TI, Kurniawan A (2020). Statin therapy did not improve the in-hospital outcome of coronavirus disease 2019 (COVID-19) infection. Diabetes Metab Syndr.

[96] Tritschler T, Kraaijpoel N, Le Gal G, Wells PS (2018). Venous Thromboembolism: Advances in Diagnosis and Treatment. JAMA.

[97] Glynn RJ, Danielson E, Fonseca FA, Genest J, Gotto AM, Kastelein JJ (2009). A randomized trial of rosuvastatin in the prevention of venous thromboembolism. N Engl J Med.

[98] Stewart LK, Sarmiento EJ, Kline JA (2020). Statin Use is Associated with Reduced Risk of Recurrence in Patients with Venous Thromboembolism. Am J Med.

[99] Agarwal V, Phung OJ, Tongbram V, Bhardwaj A, Coleman CI (2010). Statin use and the prevention of venous thromboembolism: a meta-analysis. Int J Clin Pract.

[100] Kunutsor SK, Seidu S, Khunti K (2017). Statins and primary prevention of venous thromboembolism: a systematic review and meta-analysis. Lancet Haematol.

[101] Li L, Zhang P, Tian JH, Yang K (2014). Statins for primary prevention of venous thromboembolism. Cochrane Database Syst Rev.

[102] Itkonen MK, Tornio A, Lapatto-Reiniluoto O, Neuvonen M, Neuvonen PJ, Niemi M (2019). Clopidogrel Increases Dasabuvir Exposure With or Without Ritonavir, and Ritonavir Inhibits the Bioactivation of Clopidogrel. Clin Pharmacol Ther.

